# Proteomics with a pinch of salt: A cyanobacterial perspective

**DOI:** 10.1186/1746-1448-4-1

**Published:** 2008-04-15

**Authors:** Jagroop Pandhal, Phillip C Wright, Catherine A Biggs

**Affiliations:** 1 Biological and Environmental Systems Group, Department of Chemical and Process Engineering, The University of Sheffield, Mappin Street, Sheffield, S1 3JD, UK

## Abstract

Cyanobacteria are ancient life forms and have adapted to a variety of extreme environments, including high salinity. Biochemical, physiological and genetic studies have contributed to uncovering their underlying survival mechanisms, and as recent studies demonstrate, proteomics has the potential to increase our overall understanding further. To date, most salt-related cyanobacterial proteomic studies have utilised gel electrophoresis with the model organism *Synechocystis *sp. PCC6803. Moreover, focus has been on 2–4% w/v NaCl concentrations within different cellular compartments. Under these conditions, *Synechocystis *sp. PCC6803 was found to respond and adapt to salt stress through synthesis of general and specific stress proteins, altering the protein composition of extracellular layers, and re-directing control of complex central intermediary pathways. Post-transcriptional control was also predicted through non-correlating transcript level data and identification of protein isoforms.

In this paper, we also review technical developments with emphasis on improving the quality and quantity of proteomic data and overcoming the detrimental effects of salt on sample preparation and analysis. Developments in gel-free methods include protein and peptide fractionation workflows, which can increase coverage of the proteome (20% in *Synechocystis *sp. PCC6803). Quantitative techniques have also improved in accuracy, resulting in confidence in quantitation approaching or even surpassing that seen in transcriptomic techniques (better than 1.5-fold in differential expression). Furthermore, *in vivo *metabolic labelling and *de novo *protein sequencing software have improved the ability to apply proteomics to unsequenced environmental isolates. The example used in this review is a cyanobacterium isolated from a Saharan salt lake.

## Review

### 1.0 Introduction

Increasing salinity, believed to affect nearly one-fifth of the world's irrigated land, is a major factor impairing worldwide agricultural productivity [1,2]. Furthermore, the problem is predicted to get considerably worse over the next 30 to 50 years [3]. Despite the importance of this problem, compounding effects of salinity and associated environmental stresses are not fully understood, particularly at the molecular level.

Cyanobacteria, formerly referred to as blue-green algae, are oxygenic phototrophic bacteria [4] which possess an internal membrane system, the photosynthetic thylakoid membrane. They are a multifarious group of organisms known to have colonised a wide range of ecosystems including soil, air, dry rock and aquatic systems [5]. Many species are capable of not only surviving, but thriving in conditions previously thought to be inhabitable, tolerating desiccation, high temperatures, extreme pH and high salinity, illustrating their capacity to acclimate to extreme environments [6]. Cyanobacteria have been classified into three groups relating to their salt tolerance, salt sensitive (or stenohaline), moderately halotolerant, and extremely halotolerant [7]. Example genera for each group include *Anabaena*,* Synechocystis *and *Aphanothece*, respectively.

Understanding how cyanobacteria acclimate to saline environments has been a source of interest, fuelled by two main drivers, agriculture and biotechnology. Firstly, comparable to higher plants, cyanobacteria perform oxygen evolving photosynthesis, and therefore they share many functional pathways. It is relatively easy to culture many cyanobacterial species and additional advantages such as rapid growth rate and low culture costs make them ideal models for investigating metabolic processes such as photosynthesis and respiration. Cyanobacteria therefore make suitable models for studying the physiology of salt tolerance and this has provided valuable insights into revealing the nature in which salinity prevents crop plant species from using aquatic resources [3,8].

From a non-agricultural perspective, some of the osmotic compounds produced in response to high salt have the potential to play important roles in biotechnology and medicine. Osmotic compounds (also known as compatible solutes) are low-molecular mass, uncharged, hydrophilic molecules that do not interfere with cell metabolism. These compatible solutes help restore osmotic balance with surroundings and maintain membrane integrity and protein stability [9,10]. Cyanobacteria are able to synthesise their own compatible solutes, as well as uptake them from the surroundings. They help stabilise and even enhance protein activity, for example, polyethyl glycols improving crystallization of proteins [11]. In medicine, trimethylamine N-oxide has the capability of *in vitro *rescue of cystic fibrosis (misfolded) proteins [12]. They are also used to protect cells from stress in the cosmetics industry, for example, ectoine has been shown to protect skin from UVA-induced damage [13,14].

Biochemical, physiological and genetic studies have revealed how cyanobacteria are able to adapt to high salt environments, initially by active ion export followed by accumulation of compatible solutes [5,15,16]. However, the discovery of salt-regulated genes in cyanobacteria has been accelerated by recent progress in functional and structural genomics. The genomes of over 28 cyanobacteria are now fully sequenced (with at least 72 in progress) according to the Genomes On-Line Database [17] allowing more directed analysis on an individual gene scale as well as on a genome scale. The majority of these more global functional studies have been conducted on the model cyanobacterium *Synechocystis *sp. PCC6803 (henceforth referred to as *Synechocystis*). This unicellular and freshwater cyanobacterium has a fully sequenced and annotated genome [18]. It is also able to grow photoheterotrophically and is naturally transformable, all characteristics which enhance its suitability for combined systems level genetic, proteomic and metabolomic (metabolic flux) studies [18-20].

Salt studies in cyanobacteria have generated a large amount of information and are generally centred on four main thematic areas-

(1) Biochemical and physiology based studies [21-30].

(2) Salt intake and cell signalling [31,32].

(3) Gene level responses- salt regulated genes [33,34], microarrays [35,36] and mutational analysis of salt tolerance determinants [37].

(4) Post-genomics [38-41].

This work has increased our understanding of the strategies cyanobacteria implement to adapt to high salinity, and a schematic overview is given in a review by Joset *et al*. [5]. The immediate, or shock, responses include the accumulation of compatible solutes and active export of inorganic ions, with these being regarded as largely protein synthesis independent [5]. However, in order to survive in high salt for extended periods of time, cells must adapt to the new conditions. Adaptation is a long-term process, and is therefore protein synthesis dependant. It is therefore imperative to study how proteins play functional roles in salt adaptation strategies, and this review will emphasise work specifically carried out in the proteomics field.

The proteome of an organism refers to the total set of proteins encoded by its genome (protein coding genes) [42], and therefore the field of proteomics encompasses studying these proteins, specifically the change in abundance (and status of post-translational modifications (PTM's)) in cells, tissues and organelles in response to changing environmental factors. Unlike previous protein based studies, proteomics involves the global examination of proteins. This field has the capability to reveal information on the level of protein expression, protein isoforms produced from each gene, the extent to which proteins are post-translationally modified and also the cellular and sub-cellular distribution of proteins.

In the 1990's, during the advent of genetic sequencing and bioinformatics, the ability to identify proteins increased through the use of mass spectrometry (MS). Now the technique is coupled to cross examination of databases, and has become an intrinsic part of proteomics research. With higher sensitivity and throughput, the number of proteins identified per experiment has increased. Proteins can be identified by peptide mass fingerprinting (PMF) or tandem MS, and together with the technical development of analysis software, the identification of proteins has become rapid and more accurate [43]. This provides a valuable tool for investigating protein profile changes in response to environmental stimuli, such as high salt. Furthermore, it has been demonstrated that expression patterns between mRNA and proteins levels may not correlate, and that protein levels are dependant on translational controls and regulated degradation, as well as the abundance of corresponding messages (mRNA) [44,45]. These studies indicate gaps in our knowledge of how transcription, translation and post-translational modifications interact and ultimately effect protein abundance and function. By mapping which proteins interact together and which proteins play pivotal roles in certain conditions, a further understanding of survival traits, including modifications of specific cellular pathways, can be achieved. However, the task is not simple, and studies using nuclear magnetic resonance imaging have shown that proteins are more active and dynamic than initially predicted, adding complexity to understanding where each protein is located in a cell, when the protein is present and for how long, and with which other proteins it is interacting [46].

In this review, a general overview of current proteomic methodologies will be discussed, emphasising the aspect of using and analysing samples with potentially detrimental amounts of salt. Use of these technologies with the well studied model system *Synechocystis*, with associated findings will also be considered, highlighting current challenges. With the rapid advances in proteomic techniques, some of these goals will be addressed, with reference to more challenging systems.

### 2.0 Current proteomics methodologies

#### 2.1 General overview

**Figure 1:**
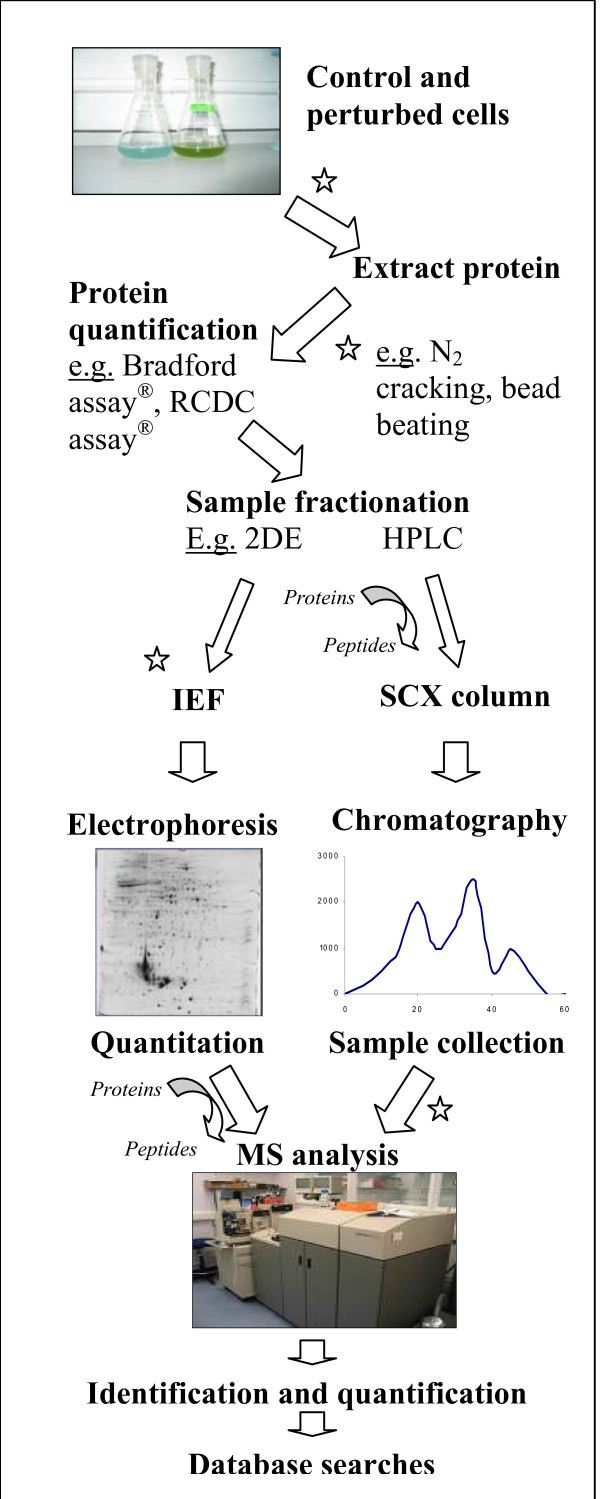
A general proteomic workflow. The main steps are, cell culture, protein extraction (liquid nitrogen freezing and cracking), sample fractionation (traditional 2DE and gel-free chromatography method), mass spectrometer analysis and protein quantitation and identification. Quantitation using 2DE is densitometry based and implemented with appropriate software (e.g. PD QUEST^®^, SameSpots^®^), but can also be undertaken on the mass spectrometer, relying on abundance of peptide peaks. Identification occurs with database search software (based on peptide mass fingerprint or sequence analysis). Proteins are commonly digested using trypsin.  : indicate essential salt removal stages. RCDC^® ^– protein quantitation assay kit, IEF- isoelectric focusing, HPLC- High Performance Liquid Chromatography, SCX- Strong Cation Exchange.

A proteomics experiment involves collaboration of several techniques, commencing with protein extraction and ending with accurate and reliable identification and quantitation. A typical proteomic workflow is presented in Figure 1.

Cyanobacterial proteomes (in common with most proteomes) are very complex, and in some cases consist of several thousand proteins [18,47]. Due to this complexity, two-dimensional polyacrylamide gel electrophoresis (2DE) has been widely utilised as the standard protein separation technique. Highly sensitive 2DE was developed in 1975, and the technique enabled fractionation of a complex mixture of proteins based on their isoelectric point (pI) and molecular weight [48,49]. The method has been shown to have very impressive resolving power [50], and allows for a quantitative comparison of protein changes in multiple samples by using a variety of staining techniques. Another major advantage of 2DE is the ability to detect protein isoforms, for example, PTM's such as phosphorylation [51,52]. However, 2DE has been criticised for a lack of automation and is often considered to be a laborious process. In addition, the success of the method is dependant on optimisation [53]. The ability to improve results by varying conditions and techniques, allows at least a satisfactory global overview of a proteome.

Recent advances in 2DE include the introduction of narrow range immobilised pH gradient (IPG) strips, which have significantly improved protein coverage [54]. IPG strips are prepared by co-polymerising acrylamide monomers with acrylamide derivatives which contain carboxylic and tertiary amino groups. The buffering groups form a fixed pH in the strip [55]. Automated spot excision systems and mass in-gel trypsin digestion techniques have also reduced the arduous nature of preparing samples from 2DE gels for MS analysis [56,57], and software packages such as Samespots and Progenesis (Nonlinear Dynamics, Newcastle, U.K.) have enhanced gel analysis capabilities. These and additional methods are extensively reviewed elsewhere [58-61]. In addition to advances in protein identification and throughput efficiency of 2DE, quantitation methodology has also improved. Fluorescent and radioactive stains can be used prior to protein separation, as well as highly sensitive post-separation stains such as SYPRO ruby, coomassie and silver stains [62-64].

Protein mixtures can now be multiplexed and run on the same gel in a technique called differential in gel electrophoresis (DIGE) [65]. Through the use of multiple fluorescent dyes to label protein samples prior to 2DE, DIGE allows for multiple samples to be co-separated and visualized on one single gel. By using CyDyes (GE Healthcare, Buckinghamshire, U.K.) not only is the sensitivity and reproducibility enhanced, but also an internal standard is included increasing statistical confidence. Unlu *et al*. [65] initially reviewed this method comparing two Drosophila embryo extracts. DIGE was found to be reproducible and sensitive, detecting differential abundance of proteins at nanogram levels.

Quantitation can also be improved by using *in vivo *isotope labelling of proteins prior to 2DE separation. This method is particularly attractive because the isotope label is incorporated into the organisms' proteome, with no physiochemical or biological consequences. Potentially all proteins are labelled, and this technique is very applicable and compatible with accompanying techniques. The different peptide isotopes can be resolved by MS, and this is where quantitation occurs (based on peptide peak areas), in contrast to staining intensity analysis, which is only semi-quantitative, and requires many replicate gels for statistical relevance. Methods include stable isotope labelling with amino acids in cell culture (SILAC) [66] and elemental labelling [67]. In the SILAC method, cell lines are cultured in media lacking an essential amino acid, supplemented with an isotopically labelled form of that amino acid, for example, deuterated leucine. This process allows quantitation of potentially all leucine containing peptides [66]. For a more universal labelling method where all peptides can be labelled, elemental labelling with media containing ^15^N or ^13^C isotopes can be performed [67]. As demonstrated by Snijders *et al*. [68], combining metabolic labelling with gel electrophoresis means a further step is added to the traditional method. However, this produces a faster workflow to identify differentially expressed proteins, as the staining intensity is used as an initial pre-screen for differentially stained protein spots, and only these are selected for identification and accurate MS-based quantitation [68]. Two gels are run in parallel with each containing proteins sourced from two phenotypes, with one isotopically labelled reference phenotype (the reference phenotype is the same in both gels). A comparison of staining intensity can then highlight potentially differentially expressed proteins. In contrast to a traditional 2DE workflow, each spot potentially represents proteins from two phenotypes. Corresponding protein spots from both gels are excised and trypsin digested, and upon resolving peptides by MS, relative ion intensity from both phenotypes can be used for accurate quantitation. Another advantage of this method is that two phenotypes can be combined in one gel (labelled and un-labelled) reducing technical variation [68].

In recent years, developments have been made to replace gel-based fractionation to overcome their limitations. Complex protein mixtures can be separated based on physical and chemical properties, including size and hydrophobicity [69,70]. It is also possible to digest proteins into peptides and separate on peptide properties [71-73]. Quantitation using non-gel-based (shotgun) methods has progressed rapidly, and usually involves comparison against an included standard. Quantitation can either be relative or absolute, and example methods are discussed further in section 4.

The common link in all the techniques discussed above is the mass spectrometer. It plays an important role in protein identification and can also be implemented for quantitation. Principally the mass spectrometer measures the mass-to-charge ratio of analytes (peptides). Analytes measured in the gas phase are ionised most popularly by electrospray ionisation (ESI) or matrix-assisted laser-desorption ionization time of flight mass spectrometry (MALDI-TOF MS) [74,75]. MALDI-MS is usually coupled to time-of-flight (TOF) analysers and therefore intact peptide masses are measured, and these are subsequently matched to theoretical masses in a database to provide protein identification (known as PMF). ESI is mostly coupled to ion traps, triple quadrupole or quadrupole-TOF instruments, which fragment selected precursor ions (peptides) further and generate ion spectra. Utilising an array of different algorithms, this information is matched against protein sequence databases to provide protein identifications and information on peptide sequence is also provided. Aebersold *et al*. [76] presents a general insight into mass-spectrometry-based proteomics. Dealing with the extensive amount of data generated from these global scale studies is a challenge, and the role of bioinformatics in proteomics is fundamental. A review of bioinformatics in proteomics with particular reference to the development of novel algorithms and discussion of the integration of bioinformatic resources can be found in Blueggel *et al*. [77] and Huang *et al*. [78].

#### 2.2 Working with 'Salty' samples

Salt poses a serious problem for resolving proteins by 2DE, but particularly in the first dimension where high voltage is required to focus proteins by isoelectric point. Removing salt is therefore imperative to allow sufficient focusing, minimise sample loss and avoid poor resolution due to streaking on gels. Salt removal can be performed during several stages of the general proteomic work-flow as summarised in Figure 1.

##### 2.2.1 Salt removal before protein extraction

Cell harvesting requires centrifugation where the supernatant (media) is removed, leaving a cell pellet. To remove external salts, several further wash steps are easily performed by resuspending the pellet in a non-saline solution, for example growth media with no added salts, and repeating the centrifugation. In halophiles, cells lyse when the environmental salt concentration drops below ca. 10–15%. Therefore salt cannot be removed with a wash step here and instead suspension of cells in a low-salt solution can be used directly for protein extraction by inducing osmotic shock. However, after protein recovery from the supernatant by high speed centrifugation (250,000 × g), a wash step can be incorporated to remove excess salt.

##### 2.2.2 Salt removal after protein extraction

At the Korea University, Seoul, the use of 3 kDa molecular weight (M_r_) cut-off columns was successfully utilised to remove excess salts from proteins [79]. These filtration columns contain pores in which only small molecules, for example, water and salt can enter, excluding proteins. The nature in which the desalted protein sample is collected (i.e. to avoid any protein loss) means that extra elution buffer is present but this dilution of the protein sample is relatively small. Another method that has gained popularity is dialysis using microdialysis tubes and centrifugation. The result is a marked improvement in protein resolving power but the time added to the overall method can be two and twelve hours, a definite disadvantage as some proteases may still be active during this period [53]. This process requires a lot of solution, whereas small volumes of protein solution are more commonly used in proteomic methodologies. Another disadvantage is that some proteins can precipitate after dialysis [80].

Ultrafiltration is not a widely used technique, but has been successfully implemented to remove salt [81]. The ultrafiltration device is fitted with a filter membrane which usually allows molecules smaller than 5 kDa to pass through, but retain larger molecules in the cell such as proteins, when high pressure is applied. This method has the benefit of not increasing the sample volume.

Precipitation methods have been included in the proteomics process for several reasons, mostly to concentrate highly dilute samples, inhibit protease activity and again to remove disruptive material such as salt. Recently, Smith *et al*. [80] compared several precipitation methods using trichloroacetic acid (TCA), acetone, chloroform/methanol, ammonium sulphate as well as ultrafiltration. The most promising results were observed using TCA and ultrafiltration due to the advantageous desalting effects. The TCA process is very easily applied, consisting of two main steps, incubation at -20°C with TCA/acetone, and removal by washing with TCA and centrifugation [82]. However, it was reported that TCA precipitation results in integral membrane proteins loss, highlighting the need for alternative methods when resolving membrane proteins by electrophoresis [83]. Finally, precipitation with ammonium sulphate was shown to be useful when attempting to eliminate high abundance proteins [80].

##### 2.2.3 Salt removal during fractionation

It is possible to adjust parameters or commonly used methods so as to eliminate interfering salt. Increasingly popular is the incorporation of a desalting step in the first dimension of a 2DE experiment, isoelectric focusing (IEF) [84]. By including an initial low-voltage step, the highly charged salt in the sample can migrate to the electrode, allowing the lesser charged proteins to migrate without interference. However, this may not be solely sufficient when working with hypersaline conditions, for example >6% w/v NaCl, and can add up to nine hours to the first dimension step. Cup-loading during IEF allows a concentration maximum of 50 mM salt, higher than passive rehydration of IPG strips [85]. In cup-loading, the protein sample is applied onto a small area of the strip during the focusing program, using a plastic cup, whereas in passive rehydration the sample and rehydration buffer are added directly to the entire strip and allowed to absorb for up to 12 hours. For passively rehydrated IPG strips, it has been shown that a simple regime of three, ten minute washes prior to focusing improves the quality of second dimension separation [86].

##### 2.2.4 Online salt removal with instrumentation

More sophisticated methods of desalting have also been developed to remove small quantities of salt prior to MS. Particularly useful for high-throughput laboratories dealing with a large number of salt samples, is the coupling of a desalting chip with ESI-MS. Proteins are adsorbed on a hydrophobic poly(vinylidene difluoride) membrane, which allows the washing out of salts [87]. Furthermore, microspin columns with modified C18 membranes have been shown to be particularly rapid at salt removal [88].

Desorption electrospray ionisation (DESI) with MS analysis has been shown to tolerate higher levels of salt. Salt is particularly problematic when using ESI, and leads to ion suppression [89]. Non-volatile materials like salts alter the efficiency of droplet formation, which in turn negatively affects the number of ions reaching the mass spectrometer. Adding ammonium acetate to the buffer in ESI is known to reduce ion suppression effects in ESI, but the ability to optimise the spray solvent and use a variety of different surfaces for initial sample analysis using DESI, allows successful analysis of samples with 0.5 to 2% salt [90].

##### 2.2.5 A simple salt removal strategy

**Figure 2:**
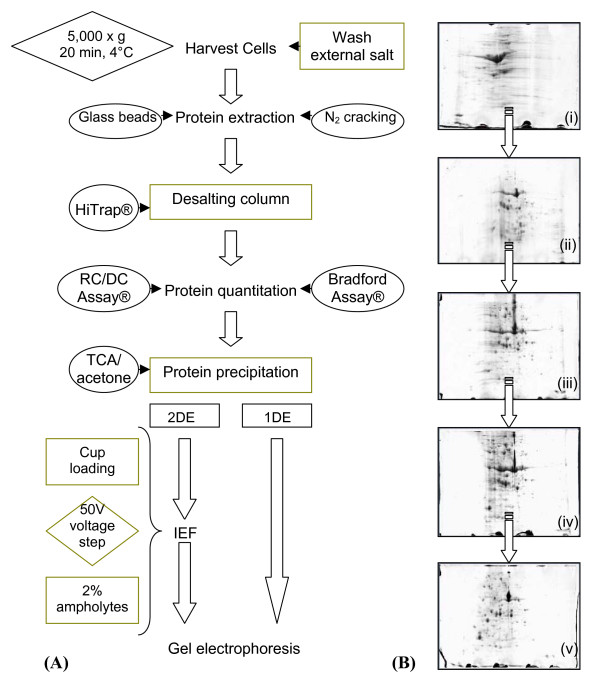
(A) A simple salt removal strategy for proteomic experiments using gel electrophoresis for protein fractionation. Desalting steps are in green boxes, example techniques in circular boxes and parameters in diamond boxes. N.B. For halophilic organisms the wash step with no salt buffer must be performed at the protein extraction stage. (B) *Euhalothece *desalting optimisation tests. 7 cm^2 ^gels, pI 3–10. i) No desalting steps ii) TCA/acetone precipitation only iii) Desalting column not used iv) External salt not washed v) Complete desalting procedure followed.

Figure 2A schematically presents a salt removal strategy when using 2DE or one-dimensional (1DE) sodium doecyl sulphate polyacrylamide gel electrophoresis (SDS-PAGE) techniques, based on the current developments discussed above. Specifically, this method is easy to implement and relatively inexpensive. When used with halotolerant organisms, the washing buffer step is performed with a non-saline buffer at the cell harvesting stage, whereas with halophilic organisms it is applied at the protein extraction stage. To demonstrate the effectiveness of this procedure, Figure 2B shows a selection of small format (7 cm^2^) 2DE gels which were run for cyanobacterial protein extracts taken from cultures grown in 6% NaCl. Four gels were run with the elimination of different salt removal steps as shown in Figure 2A, and in the final gel the full procedure was followed.

### 3.0 Proteomic salt responses in a model system

The majority of proteomic studies on salt tolerance in cyanobacteria have been conducted on *Synechocystis*, using 2DE. Newly expressed or proteins with enhanced expression in salt stressed cells have been identified, and a group of these are termed 'stress proteins'. These studies have contributed to the construction of the Cyanobase database, containing over three thousand predicted protein coding genes [91]. Many of these proteins are annotated as 'hypothetical', as sequence similarity to proteins of known function is absent. Methods for isolating plasma, thylakoid, periplasmic and outer membrane proteins have been developed, as well as isolating pure plasma and periplasmic space proteins [41,92-96]. This has allowed analysis of how proteins in different subcellular compartments assist in cellular adaptation to high salt.

#### 3.1 Salt response in different cellular compartments

**Figure 3:**
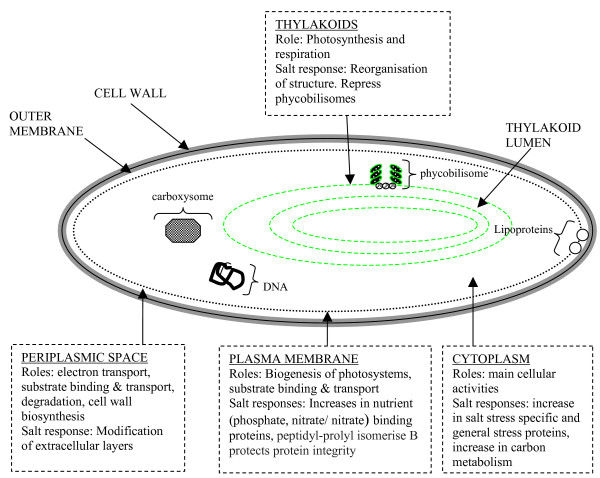
Salt responses at the protein level. A summary of responses to varying degrees of salt stress (~2–4% w/v NaCl) in the main cellular compartments of a unicellular cyanobacterium cell, as revealed by proteomics studies [[38,39,41,109]].

Figure 3 summarises the proteomics-based responses to salt stress in a unicellular cyanobacterial cell, and Table 1 defines the corresponding conditions under which cells were investigated. Cells were grown in BG11 media [97] supplemented with NaCl unless otherwise stated.

**Table 1:** A summary of proteomic studies in cyanobacteria.

Organism	NaCl concentration	Incubation period	Subcellular fraction	Additional growth conditions*	Reference
*Synechocystis *sp. PCC6803	342 mM or ~2%	6 days	Periplasmic	29°C, 170 mmol photons m^-2 ^s^-1^	[41]
*Synechocystis *sp. PCC6803	342 mM or ~2%	6 days	Periplasmic	29°C, 170 mmol photons m^-2 ^s^-1^	[38]
*Synechocystis *sp. PCC6803	400 mM or ~2.4%	Several generations	Membranes	32°C, 50 mmol photons m^-2 ^s^-1^	[96]
*Spirulina platensis*	800 mM or ~4.8%	n/a	Thylakoids	30°C, 80 mmol photons m^-2 ^s^-1^	[109]
*Synechocystis *sp. PCC6803	684 mM or ~4%	6–8 days	Plasma	30°C, 80 mmol photons m^-2 ^s^-1^	[102]
*Synechocystis *sp. PCC6803	684 mM or ~4%	5 days	Cytoplasmic	30°C, 170 mmol photons m^-2 ^s^-1^	[39]

The conditions and subcellular compartments investigated are defined and the studies are listed in chronological order.

* Temperature and light conditions.

##### 3.1.1 Periplasmic Space

The periplasmic space (Figure 3) is situated between the plasma membrane and the outer membrane. No active ion transport mechanisms have been found in the outer membrane in *Synechocystis *cells, but transport proteins involved in the uptake of substrates are present in the periplasmic space, and these could be affected by high salt levels. This inspired Fulda *et al*. [41] to use a cold-osmotic shock technique [98] to isolate and compare the abundance of periplasmic proteins from *Synechocystis *grown in low salt (2 mM or 0.01% NaCl) and high salt (342 mM or 2% NaCl). The method of periplasmic protein isolation is unsuitable for examining adaptation to higher salinities, as the sample fraction becomes contaminated with proteins from other cellular compartments in these conditions. Extracted protein fractions were separated by SDS-PAGE, and two bands of interest were subjected to N-terminal sequencing. Unfortunately, both identified proteins were annotated as hypothetical [41].

By implementing 2DE for enhanced resolution with MALDI-TOF MS, the experiment was repeated [38]. pH 4–7 IPG strips were used with 23 × 23 cm 12.5% acrylamide gels. MALDI-TOF MS allows protein identification by soft ionisation methods with a TOF mass spectrometer [75]. Protein fragments generated by protease digestion are fixed in a solid phase matrix and ionised by laser. TOF analysis reveals experimental peptide masses which can then be matched to theoretical masses in a database. Six and three proteins were identified as enhanced and newly expressed, respectively, in the presence of elevated levels of salt [38]. Several of these proteins were thought to be involved in synthesising or modifying extracellular layers in *Synechocystis*, highlighting the importance of changes in cyanobacterial cell walls in adapting to high salinity. For example, a protein showed significant similarity to phosphoglycerate mutases from *Streptococci*, which are involved in polysaccharide synthesis in their capsules [38]. Furthermore, a salt enhanced protein was found to be homologous to cell surface lipoproteins of *Mycobacteria *spp. [38].

It is apparent from this study, that functional annotation of *Synechocystis *is still at an early stage, and characterisation of these salt enhanced proteins required searching for similar functional domains present in the databases for other organisms. A number of periplasmic proteins were also observed to have reduced expression in saline media, and these were attributed to the expected reduction in protein synthesis of non-stress proteins in salt stressed cells [38,99].

##### 3.1.2 Membrane Proteins

A combination of blue native/SDS-PAGE and MALDI-TOF was used to study the composition and dynamics of membrane protein complexes in *Synechocystis*, perturbed by three different growth modes and varying levels of CO_2_, iron, or salt [96]. Blue native is a proteomic technique implemented in order to separate and study intact protein complexes at high resolution [100,101]. It enables further elucidation of protein function by analysing protein-protein interactions. A total of 53 protein spots were identified in this study, corresponding to 37 different *Synechocystis *genes [96]. A stress of 0.4 M (~2.4%) NaCl was applied to cells for 72 hours, but this did not induce any observable changes in the membrane protein complexes, which agreed with previous physiological studies (reviewed by Joset *et al*. [5]) that the plasma membrane and cytoplasm are likely to be the main subcellular compartments affected by salinity.

Huang *et al*. [102] screened for proteomic changes in the plasma membranes of *Synechocystis *in response to salt. The plasma membrane represents a barrier to the surrounding medium, and is likely to be responsive to environmental salinity changes [38]. These membranes were isolated by sucrose density gradient centrifugation and two-phase partitioning [92]. 106 proteins were identified, corresponding to 66 gene products, using 2DE and MALDI-TOF MS. 25 proteins changed significantly in abundance due to salt concentrations of 684 mM (~4.1%). A methanol/chloroform precipitation step was included after protein extraction to desalt and concentrate the sample [103]. An advantage of using 2DE for protein separation and visualisation is particularly apparent in this study, as isoform spots were clearly visible. Abundance changes in some of the isoforms imply PTM's may play a role in protein function in the stress conditions. Nearly one third of the salt-enhanced proteins identified in this study are substrate binding ABC transporters [102]. An increase in glucosylglycerol-binding protein, GgtB, was expected since *Synechocystis *cells can uptake this compatible solute from its environment. The evidence for an interrelation between salt and iron-stress in this organism was supported by the very high accumulation of iron binding lipoprotein, FutA1. It has been previously proposed that FutA1 plays a protective role in photosystem II under conditions of iron deficiency [104]. Increases in phosphate and nitrite/nitrate binding proteins have been hypothesised as a necessity for cells to overcome salt induced nutrient deficiency, a problem resulting from plasma membrane structural changes [105]. Moreover, an increase in the regulatory protein PII suggests a change in the carbon and nitrogen balance in salt-stressed cells.

Further salt-induced proteins, thought to play significant roles in stress, included vesicle-inducing protein in plastids (Vipp1), a protein involved in thylakoid membrane biogenesis in *Arabidopsis *[106], membrane-bound peptidyl-prolyl isomerase B, which could be involved in maintaining the integrity of proteins in the plasma membrane and CoxB, which supports recent work suggesting a role in managing photosynthesis in stressed cells [39]. Some of the hypothesised functions in salt tolerance require further characterisation, at the protein level through, for example, structural studies, or at the gene level via knock-out mutations from the protein-coding genes and assessment of phenotypic alterations. This is particularly true for hypothetical proteins which have no associated function. In addition to salt regulated proteins, 21 proteins were newly identified [102] and before further studies are undertaken it is possible to predict function to a certain limit. A plethora of open source software tools have been developed, with algorithms which help to predict cellular functions from conserved domains. Furthermore, characteristics such as hydrophobicity and attributes like lipoprotein or sec- signals, can be used to predict subcellular localisation [107,108].

Many proteins from the plasma membrane were associated with salt stress in *Synechocystis *for the first time in this study, however, the limitations of using 2DE were recognized by virtue of the fact that no integral membrane proteins were identified [102].

##### 3.1.3 Cytoplasm

Fulda *et al*. [39] used 2DE with MALDI-TOF to investigate the soluble proteome response in *Synechocystis *cells to approximately 4% salt, and 55 out of the 337 identified proteins were induced. In the salt-acclimated cells, induced proteins were organised into four groups; stress proteins which are salt specific, general stress proteins, enzymes involved in basic carbon metabolism and hypothetical proteins.

Specific salt stress proteins included the enzyme ADP-glucose pyrophosphorylase, responsible for synthesising the precursor ADP-glucose for the compatible solute glucosylglycerol. General stress proteins included molecular chaperones GroEL1 and elongation factor-Tu. Increased amounts of soluble electron carriers, flavodoxin and plastocyanin were also observed, and these are thought to play an important role in adjusting to stress-induced changes in electron transfer [39]. Several enzymes involved in carbohydrate metabolism also accumulated, including transketolase, glycogen phosphorylase and phosphoglycerate kinase [39]. The accumulation of the glucosylglycerol is proposed to cause this change in carbon metabolism.

##### 3.1.4 Thylakoids

A method for isolating pure thylakoid membrane proteins suitable for 2DE analysis was developed nearly a decade ago, implementing aqueous polymer two-phase partitioning in combination with sucrose density centrifugation [92]. Despite this, a large scale proteomics assessment of the salt response of the photosynthetic membranes has not been performed. By combining traditional protein separation and quantitation methods SDS-PAGE and western blotting, Sudhir *et al*. [109] investigated the response of thylakoid membrane proteins in the cyanobacterium *Spirulina platensis *to 0.8 M (~4.8%) NaCl. *S. platensis *was isolated from sodium (150–200 mM Na^+^) rich lakes and ponds and is cultured in Zarrouk medium [109]. A 40% decrease in protein D1 from the PSII reaction centre was observed. Additional abundance changes in proteins were observed but these were not identified. The authors undertook additional physiological studies and postulated that a variety of effects occurred on photosynthetic electron transport activities due to the marked alterations in the composition of thylakoid membrane proteins [109].

Vipp1 protein, (see section *3.1.2*), is located in the plasma membranes, but has been associated with thylakoid membrane formation in *Arabidopsis *[106]. It shares sequence similarity to stress related phage shock protein PspA [106], and a knockout mutant created in *Synechocystis *led to an inability to form thylakoid membranes. An increase in this protein in salt acclimated cells may imply a structural reorganisation in these membranes is necessary [102].

#### 3.2 Confirmation methods

In these studies, a confirmation technique is often used in parallel with newly developed proteomics workflows with the aim of confirming their reliability and applicability. Complementary techniques for validation of protein abundance changes have recently been incorporated into many proteomic studies, with western blotting being the most widely applied [110]. This practice is normally encouraged for several proteins. For example, immunoblotting analysis confirmed the expression changes of several proteins in the study by Huang *et al*. [102].

#### 3.3 Shock versus adaptation

Biological function is predominantly facilitated by proteins, highlighting the power of proteomics to identify the physiological changes which take place in cells in order to survive adverse conditions. However, transcriptomics is useful for assessing immediate gene expression changes, and therefore integrating both sets of data is necessary for a more complete systems level understanding. Nevertheless, proteomics can yield insight into immediate and long-term physiological responses. Fulda *et al*. [39] used 2DE in a proteomics study to address the salt shock (immediate) response versus salt adaptation (long-term) response in *Synechocystis *to approximately 4% NaCl. To produce a meaningful comparison, previously generated transcriptomic data and a survey of changes in physiological parameters were used to identify the most revealing times to harvest cells. Short-term shock and long-term adaptation times were at 2 hours and 5 days, respectively [36,39]. A pulse ^35^S-methionine labelling method was used, allowing clear visualisation of newly induced proteins from radioactive 2DE-gels. *In vitro *metabolic labelling of cells with radioactively labelled amino acids is routinely performed, but despite advantages such as relatively high energy beta emitter potential and high specific activity, any proteins lacking methionine would be undetected [111,112]. Despite the fewer induced proteins identified compared to genes induced in a microarray study, 90% of these identified proteins were also induced at the transcriptional level [36].

As expected, heat shock proteins (which protect or repair proteins), DNA and RNA-binding stress proteins and antioxidative enzymes all increased in expression in the short and long-term, in both transcriptomic and proteomic studies [35,36,39,102]. However, only truncated versions of the chaperone GroEL1 and the elongation factor-Tu, were identified in the long term by proteomics, and whether this has functional significance remains unknown [39]. Seven enzymes involved in carbohydrate metabolism also accumulated after 5 days, whereas a similar pattern in gene expression was not observed. In fact, in this study, nearly half of the data for induced genes in acclimated cells (5 days) did not correlate with protein inductions [39]. Therefore, the authors proposed that PTM's may play a role in salt adaptation, particularly considering the multiple spots identified in gels corresponding to the same protein.

### 4.0 Advances in proteomic methodologies

The studies discussed in section 3 lay the foundations for understanding global cellular salt responses. However, 2DE is the technique invariably used for protein separation and quantification. Limitations of 2DE are apparent when comparing proteomic data with transcriptomic data, most significantly, coverage of the full proteome is poor. Unfortunately, low abundance proteins, proteins with extreme pIs, membrane and membrane bound proteins (due to their hydrophobicity), cannot readily be resolved by 2DE [113]. For example, identifying and studying proteins with a pI of 4 to 7, is only part of the salt adaptation story. A protein pH range of 4 to 7 is popular for studying *Synechocystis*, as its predicted proteome is biased towards an acidic pH [17]. Furthermore, the presence of high abundance pigment proteins, for example, phycocyanin proteins in cyanobacteria, can cause smearing in gels and reduce the quality of resolution and quantitation [39]. In addition to proteome coverage issues, protein abundance changes based on staining intensity are only semi-quantitative. Consequently, this has led to the development of gel-free proteomics techniques, sometimes referred to as shotgun proteomics.

As mentioned previously, the rapid development in shotgun proteomics has led to an array of techniques to fractionate complex protein samples. For example, proteins can be separated by characteristics such as size, hydrophobicity or charge using size exclusion chromatography (SEC) [69], reverse-phase (RP) chromatography [70] and weak/strong anion exchange chromatography (WAX/SAX) [114,115], respectively. Multi-dimensional separation schemes have also been applied at the peptide level, where proteins are digested with an appropriate enzyme, for example trypsin, and then separated by methods including strong cation exchange (SCX) [71], capillary isoelectric focusing [72] and liquid phase isoelectric focusing [73].

By combining an array of peptide and protein fractionation techniques prior to MS analysis, the proteome coverage can be increased significantly [116,117]. Gan *et al*. [118] compared a combination of six fractionation workflows employing techniques which separate proteins via IEF, 1-D PAGE, WAX chromatography, as well as peptide separation using IEF or SCX, all prior to reverse phase multi-dimensional liquid chromatography and MS analysis [118]. 776 proteins were identified in *Synechocystis*, representing over 20% of the total predicted proteome. In conjunction with technical advances in this area, the need to increase the representation of an organism's proteome continues. One challenge is to increase the solubility of membrane proteins whilst maintaining compatibility with downstream processes [119,120]. This can be achieved through the use of high percentage organic solvents and acids. Moreover, the use of strong zwitterionic and non-ionic detergents have been shown to improve the solubilisation of membrane proteins from cyanobacteria [121]. In a study conducted on the filamentous cyanobacterium, *Anabaena variabilis*, such techniques led to the identification of 646 (13%) proteins [121].

With the ability to identify hundreds of proteins in a single proteomics experiment, the need to quantify differential protein abundance efficiently and accurately, has been realised. It is now possible to perform a large scale proteome study by identifying and quantifying proteins from complex samples. Quantitation using shotgun methods has progressed at an impressive rate utilising isotope labelling strategies such as SILAC. Isotope tags can also be added enzymatically by transferring ^18^O to peptides from water [122]. In addition, chemical reactions are used where reagents are tagged to the protein or peptides, and these provide internal standards for quantitation. Examples include isotope coded affinity tags (ICAT) [123], mass coded abundance tagging (MCAT) [124], the AQUA strategy (for absolute quantitation) [125], and isobaric tags for relative and absolute quantification (iTRAQ) [126].

The iTRAQ procedure has recently gained in popularity [127-130]. It is usually based on a peptide labelling strategy, where four or eight amine reactive isobaric reagents are used, enabling the comparison of several phenotypes in one experiment (including technical and biological replicates). The quantification occurs at the MS/MS stage, and thus requires a tandem mass spectrometer [131]. MS/MS leads to fragmentation of peptides into individual amino acids allowing a far more accurate sample analyses.

**Figure 4:**
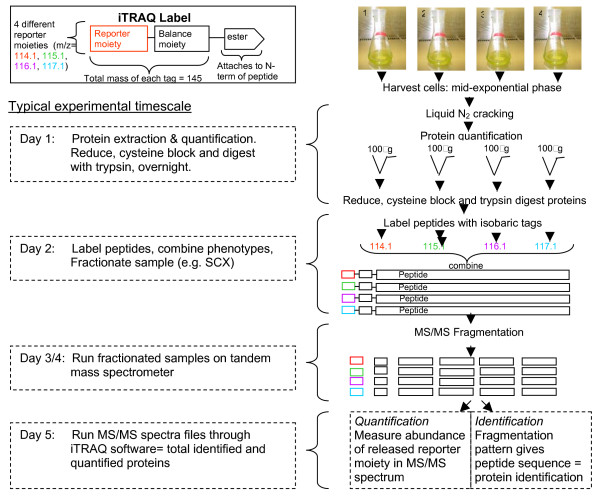
An overview of a proteomic procedure with iTRAQ reagents. Proteins are extracted from four phenotypes which are to be compared and trypsin digested. The resulting peptides are labelled with isobaric tags and then pooled for fractionation and analysis at the MS/MS stage on the mass spectrometer. Isobaric tag intensity corresponds to peptide abundance and is used for protein quantitation.

iTRAQ has been demonstrated as a useful, robust and reliable tool for proteomics [131], with several advantages over densitometry and other labelling techniques including quantitation accuracy and overall workflow efficiency. A typical iTRAQ experiment takes no more than several hours over approximately 2 to 3 days to label and pre-process the samples, 2 to 3 days (based on 20 fractions × 135 minutes runs) to separate and analyse by MS, and 1 day to initially process the MS data (Figure 4). Moreover, these runs would include biological and technical replicates [129]. This time-scale and work load is considerably less than a 2DE work-flow which requires running 4 separate gels (in triplicate). Each gel requires 1^st ^dimension separation (day 1), 2^nd ^dimension separation (day 2), staining (overnight), spot matching and identifying spots of interest, cutting all spots (approximately 500–1000) and tryptically digesting (at least several days), peptide extractions (several days), and finally MS analysis (several days).

Choe *et al*. [132] demonstrated the consistency of observations using 2DE and iTRAQ, as well as the reproducibility of the experiments. They reported that half of the quantified protein expression ratios have a coefficient of variation (CV) less than 0.31 using 2DE and less than 0.24 using isobaric tags; whereas 95% of the quantified protein expression ratios have a CV less than 0.81 using 2DE and less than 0.53 using isobaric tags [132]. Wu *et al*. [133] compared iTRAQ to DIGE and ICAT, and reported its superior quantitative sensitivity. A survey of technical, experimental and biological variations using the technique was also undertaken using *Synechocystis *as a model organism [129]. Furthermore, Chong *et al*. [128] used iTRAQ with *Synechocystis*, demonstrating the use of a multiple injections strategy to improve proteome coverage. An average of 218 proteins was identified and the issue of abundant pigment proteins was highlighted. There have been no studies to date, looking at salt stress or adaptation in cyanobacteria, using shotgun proteomic techniques.

### 5.0 Challenging systems

*Synechocystis *is a model organism for photosynthesis research and has therefore been investigated extensively. It is also the model organism for cyanobacterial and higher plant salt stress studies, and this is the case in proteomics. Its genome is fully sequenced which makes identification of proteins through MS and database searching, relatively simple. The expectations are that discoveries made in salt stress proteomics studies in *Synechocystis*, will provide insight into the adaptive mechanisms in other organisms. Uncovering the proteomic response of this model organism has made an invaluable contribution to understanding salt acclimation. However, cyanobacteria are ancient life forms and evolutionary pressures have created many diverse species, including those which can survive in higher salt concentrations. Unfortunately, the majority of these cyanobacteria do not have their genomes sequenced, so how is it possible to identify proteins and hence study the proteome of an unsequenced organism? This is an area known as cross-species proteomics, and is yet to be fully exploited [134,135].

#### 5.1 Cross species proteomics

Accompanying the rapid growth in the number of fully sequenced genomes, is the increasing number of protein sequences present in databases. This maximises the chance of identifying proteins from a non-sequenced organism using conventional protein identification software. Conventional software, for example Mascot (Matrix Science, London, UK) relies on matching of tryptic peptides to theoretical peptides present in databases. With increasing numbers of sequenced proteins, the chance of an unsequenced organism producing an exact same tryptic peptide is correspondingly enhanced. Developments in the area of MS and bioinformatics have also significantly expanded the applicability of cross-species proteomics [134-138]. Mass spectrometry-driven BLAST (MS BLAST) is a database search protocol which uses a list of peptide sequences generated by interpretation of MS/MS spectra using ProBLAST software [135,139]. Significant hits in MS BLAST are colour-coded based on a high scoring pairs (HSP's) algorithm. More recently, open source software SPIDER uses an alternative algorithm for identification of novel proteins [140]. Collectively, they rely on the interpretation of MS/MS spectra using *de novo *sequencing, a technique which has also advanced with new powerful software, for example PEAKS and OLAV [141,142].

The main challenge in cross-species proteomics is to produce high confidence protein identifications, and this requires meeting set criteria. Using probability calculations and cut-off scores in specific analysis software and fulfilling the guidelines generated for identification (I.D.) methods in proteomics journals [143], for example, requiring 2 or more peptides per protein, should be included. One approach is to design degenerate nucleotide primers for the protein coding gene by using peptide sequences detected in the MS. Alternatively, alignment files generated from nucleotide sequences from related organisms, which are present in nucleotide databases, can be used. Successful sequencing of the gene provides verification of the protein identification, and therefore extra confidence in the identification methods implemented. A method using metabolic labelling combined with gel electrophoresis [68,144] is particularly advantageous. This method was initially designed to provide a fast and accurate method for protein quantification, however, it presents the opportunity to verify protein I.D.'s for non-sequenced organisms. In this method, proteins are metabolically labelled by culturing in the presence of a heavy isotope, for example, ^13^C or ^15^N. Cyanobacteria are often cultured in BG11 media [97], and the main source of nitrogen in this media is sodium nitrate, and therefore replacing this with an isotopic Na^15^NO_3 _is relatively simple, as long as a sufficient number of generation times are included for maximum incorporation efficiency (and it is confirmed that the organism cannot fix atmospheric nitrogen). These light (^14^N) and heavy (^15^N) peptides can be distinguished by mass-to-charge scale on the mass spectrometer, and the ratio in peak height or area between the two forms correlates with protein abundance. However, the difference in mass between labelled and unlabelled versions of the same peptide provides extra identification evidence, via imposition of an elemental constraint [145].

Protein fractionation is important to reduce sample complexity, and thereby allowing more successful interpretation of meaningful MS spectra, due to issues involved with ion suppression and duty cycle [89,146]. 2DE can be used for protein separation which allows a further identification confirmation by matching gel positioning with predicted isoelectric point and molecular weight. Using highly saline samples with 2DE would require a salt removal workflow as illustrated in Figure 2. Similarly, SDS-PAGE allows confirmation by molecular weight, but has the added advantage of improved resolution of hydrophobic proteins [144]. A work-flow has been developed very recently to increase protein identifications in non-sequenced organisms using complex samples with high-throughput methods such as iTRAQ, in which case protein fractionation via gel electrophoresis is eliminated [147].

It has been demonstrated by comparative genomics [148] that the difference in salt tolerance between glycophytic and halophytic plants is quantitative, and not necessarily qualitative. Therefore, tolerance mechanisms may be conserved amongst all plant species. This highlights the importance in studying the salt response in alternative cyanobacteria despite the likelihood that 'new' proteins will not be discovered in non-sequenced isolates and there is more chance that a comparison to existing systems will be performed.

#### 5.2 Proteomics of an unsequenced extreme halotolerant cyanobacterium

We have isolated a eurohaline cyanobacterium from a salt lake in southern Libya, in the heart of the Sahara. The salinity of the lake varies by sampling location and season, but can reach up to 16% w/v [149]. This microorganism can adapt to large variances in salt concentration, and unlike halophiles, does not require salt for survival. It therefore represents an ideal system to study cellular responses to adaptation in both high and low salt environments. This ability of adapting to a large range of salinities may actually be more difficult to achieve than the fixed salt requiring metabolism of halophiles.

Furthermore, it is an environmental isolate and not sequenced, presenting a challenge for protein identification. The method combining gel electrophoresis and metabolic labelling discussed above was used to study adaptation of this organism to the addition of 0, 3, 6 and 9% salt (NaCl) to BG-11 [144]. Proteins were identified using Mascot (relying on generating identical tryptic peptides to those present in the database) and MS BLAST, where extra proteins could be identified via *de novo *sequencing and homology searching. In total, 383 unique proteins were identified and 23 different organisms were required as identification sources (17 different cyanobacteria), allowing an in-depth analysis of its adaptive response to high and low salinity [144]. This particular cyanobacterium shares many similar salt-tolerance mechanisms to *Synechocystis*, albeit in higher salt concentrations. *Euhalothece *increases production of stress chaperones and antioxidative enzyme superoxide dismutase. There appear to be alterations to the cell wall, decreased pigment production as well as significant alterations in central intermediary metabolism, including an increase in the synthesis of compatible solutes. However, its proteome displays completely different patterns when grown in no salt (i.e. 0% NaCl added). An increase in stress related chaperones was accompanied by enhanced abundance of carbon uptake and fixation proteins and periplasmic iron binding protein. Further studies need to be undertaken to further understand this response to low salt. Proteins were also identified as salt responsive which play no noticeable role in *Synechocystis *tolerance, and these also require further characterisation.

## Conclusion

With the increasing number of genome sequencing projects being undertaken and rapid advances in proteomics techniques, the opportunity for making greater discoveries in the post-genomics field has been recognised. In effect, genomic information can be decoded into a functional protein interpretation.

Understanding the salt response in cyanobacteria will make a relevant impact on ultimately understanding the detrimental effects of salinity on crops plants. *Synechocystis *is the best characterised cyanobacterium from a salt stress perspective and this stands true in the emerging field of proteomics. Gaining a systems level understanding of the salt stress response in this cyanobacterium requires generating and interpreting quantitative proteome data. Techniques are constantly and rapidly being developed and tested to overcome current limitations, and this includes the opportunity to study more difficult and challenging systems.

*Synechocystis *alters the protein composition of extracellular layers in response to salt stress, and in particular increases expression of ABC-transporters involved in nutrient acquisition in the plasma membrane. A reorganisation of the thylakoid membranes seems likely through the identification of salt-induced Vipp1, and the increase in energy capacity using PSI and respiration is important for acclimation. Specific salt stress proteins, as well as general stress proteins play an obvious role, and large changes in carbon metabolism are seen and these may be related to the long-term production of compatible solutes. Finally, a large array of hypothetical proteins were identified as playing a crucial part in adaptation, but only a small number had associated putative functions. These proteins require further investigation.

Important advances have been made in increasing proteome coverage through novel combinations of protein and peptide fractionation techniques. By combining reliable protein identifications with accurate and reproducible quantitation data, information on hundreds of proteins can be obtained in a single experimental workflow. The use of iTRAQ technology is gaining popularity here. Finally, proteomics is no longer confined to those organisms which have their genomes partially or fully sequenced. Efforts to develop techniques and interpretation software to analyse these proteomes, is beginning to reward the scientific community by producing interesting findings on the biological behaviour of fascinating, environmentally-significant organisms.

## Competing interests

The authors declare that they have no competing interests.
